# Negative Emotion Arousal and Altruism Promoting of Online Public Stigmatization on COVID-19 Pandemic

**DOI:** 10.3389/fpsyg.2021.652140

**Published:** 2021-05-26

**Authors:** Xi Chen, Chenli Huang, Hongyun Wang, Weiming Wang, Xiangli Ni, Yujie Li

**Affiliations:** School of Business Administration and Tourism Management, Yunnan University, Kunming, China

**Keywords:** stigmatization, negative emotions, social identification, altruistic behavior, COVID-19 pandemic

## Abstract

The outbreak of COVID-19 is a public health crisis that has had a profound impact on society. Stigma is a common phenomenon in the prevalence and spread of infectious diseases. In the crisis caused by the pandemic, widespread public stigma has influenced social groups. This study explores the negative emotions arousal effect from online public stigmatization during the COVID-19 pandemic and the impact on social cooperation. We constructed a model based on the literature and tested it on a sample of 313 participants from the group being stigmatized. The results demonstrate: (1) relevance and stigma perception promote negative emotions, including anxiety, anger, and grief; (2) the arousal of anger and grief leads to a rise in the altruistic tendency within the stigmatized group; and (3) stigmatization-induced negative emotions have a complete mediating effect between perceived relevance and altruistic tendency, as well as perceived stigma and altruistic tendency. For a country and nation, external stigma will promote the group becoming more united and mutual help. One wish to pass the buck but end up helping others unintentionally. We should not simply blame others, including countries, regions, and groups under the outbreak of COVID-19, and everyone should be cautious with the words and actions in the Internet public sphere.

## Introduction

Being the target of stigmatization places individuals under great pressure (Goffman, 2009). The most obvious and frequent consequence is discrimination (Berjot and Gillet, 2011). The COVID-19 pandemic aroused stigma toward people with different social roles, such as healthcare workers, patients, and survivors of the disease, as well as residents of some districts and countries (Bagcchi, 2020). Stigmatization can have deleterious effects on individuals, such as depression, anxiety, self-contempt, and lower performance (Sheehan et al., 2017; Roseman, 2018). Entering into the Internet era, the people all over the world have been connected by information online (Castells, 1996). Social networking sites extensive application makes various views and attitudes widely spread in cyberspace (Krishnan and Hunt, 2015). Particularly when some influential people in cyberspace make comments to slander other groups, it will have a significant impact on the public sentiment (Zhao et al., 2014).

The outbreak of the pandemic has put all countries under extensive and profound pressure. At present, the COVID-19 pandemic has been prevalent in the world for more than a year. In dealing with this crisis, all countries need to play a common role in terms of sharing data (Lee et al., 2020), economic coordination (McKibbin and Vines, 2020), policy cooperation (Benvenisti, 2020), and system linkage (Brown and Susskind, 2020), among other factors. However, in these efforts, stigmatization of certain regions and groups of people has been common and usually irrational (Roberto et al., 2020). In particular, the stigma that occurs among countries has damaged their relations and has become one of the obstacles to the joint efforts to fight the pandemic. While building social impressions, stigma can be a powerful tool for those who attempt to destroy certain organizations' social images (van Spanje and Azrout, 2019). For the stigmatized, public stigmatization contaminates and damages the living environment in the society (Kurzban and Leary, 2001). In the outbreak of an epidemic, stigma often links to the improper tendency of imputation (Li et al., 2020). It leads to negative emotions among the stigmatized, including stress, anxiety, sadness, and even some physical reactions (Lee and Craft, 2002; Armour, 2007; Lillis et al., 2020).

The COVID-19 pandemic is a crisis affecting all people, putting tremendous pressure on and threatening individuals and societies. Stigmatization, including violence, targeted toward Asians has increased during this period. In the United States, cities with large numbers of infections (e.g., New York, California) have seen notable increases in discrimination (Roberto et al., 2020). Stigmatization is unfavorable to the victims and rubs salt in people's emotional wounds that exist the crisis. In particular, the online public stigmatization of a certain region or group puts emotional burdens on its people and gives rise to their negative emotions. During the COVID-19 pandemic, there have been some advances in the study of the stigmatization of specific groups and occupations (Bruns et al., 2020; Roberto et al., 2020; Taylor et al., 2020). In cyberspace, the post-truth phenomenon reflects the resonance of group emotions (McIntyre, 2018), and public stigma will provoke the emotion reactions and emotional resonance of Internet group (D'Ancona, 2017). However, there is still a lack of empirical evidence on the influence of online public stigma on the emotions of the victimized groups, as well as a lack of differential tests on the degree of influence of specific types of negative emotions. Therefore, the problems that this research explores are as follows.

*RQ1: To what extent will online public stigma lead to different negative emotions among the victim groups?*

The responses to and measures taken against stigmatization are not necessarily negative; that is, stigmatized individuals are not necessarily passive people who respond actively to identity threats, nor are they condemned to develop low self-esteem (Schmitt et al., 2014). Gross (2001) believes that there exist potential mechanism between stigma and psychology called emotional regulation, people consciously or unconsciously adopt certain strategies to change some of the components of emotional responses. Whether we consider an individual, an organization, or even a country, the subject will have some self-healing power when hurt by discrimination and stigma. For example, social cooperation is a basic mechanism for coping with threats. When facing common suffering and certain threats, people tend to cooperate to face common challenges (Jervis, 1978). Collective identities lead to a general propensity to cooperate, and reason and emotions interact to create and sustain social collective identities (Lebow, 2005). Finding ways to evolve and maintain cooperative behaviors in human society and other animal populations is one of the most important research topics in evolutionary biology and the broader social sciences (Colman, 2006). Human emotions have important and complex mechanisms for the maintenance of cooperative relationships (Fessler and Haley, 2003; Pennisi, 2009). During the COVID-19 pandemic, while examining the harm caused by stigmatization by other countries, we also verify whether there are mechanisms among individuals and societies for trying to heal its negative effects. Therefore, the current study also attempts to answer the following question:

*RQ2: Will the perception of stigma during COVID-19 and the resulting negative emotions promote the group's tendency toward social cooperation?*

In the context of the COVID-19 pandemic, this study interprets the negative impact of online public stigma on group emotions and associates individual emotions with the group's tendency toward altruism.

## Stigmatization and Emotion

Goffman (1963) proposed a basic definition of stigma in a sociological study, which reflects any physical or social attribute that devalues an individual's identity and hence disqualifies the individual from full social acceptance. Stigma exists when allows the processes to unfold, such as labeling, exclusion, discrimination, negative stereotyping, and low status power situation (Link and Phelan, 2001). Three kinds of stigma were classified: abominations of the body, blemishes of individual character, and tribal stigma through race, nation, and religion (Goffman, 1963). The consequence of stigmatization is the possibility that one will suffer from discrimination, prejudice, or unfavorable treatment (Frost, 2011). The disclosures of stigmas may hamper the relationship between individuals and the reputations of the targets within and outside of professional work contexts (Ragins, 2008). The Internet has become the biggest way to connect global information, the negative influence is exacerbated for public stigmatizations shared on the worldwide social networking sites, which are circulated among people all over the world.

Stigmatization occurs within different ranges and at different levels (e.g., individual, organization, social) (Link and Phelan, 2001; Bandura, 2004). Attribution theory has been incorporated to analysis the attributions stigmatized behaviors(Corrigan, 2000; Phelan, 2005), which is a theory states the attributions people make about the cause of an outcome influence emotions, perceptions and behavior toward the individual affected by the outcome (Weiner, 1985). Researches confirm that attributing stigma to non-subjective factor, such as genetic defects, can reduce other people's prejudices (Phelan, 2005). Furthermore, to understand the meaning of stigma more deeply, the research attentions shift from attribute characteristics to social relations, as stigma is not just because of the inherent negative characteristics, the rules of social construction and creator is the perpetrator of stigma (Frost, 2011).

In a social structure where stigmatization emerges, there exist the subject and object in the role; the subject is the person who commits stigmatization and the object is the victim of stigmatization. Some researches on stigmatization distinguish the stigmatization movement and stigmatization perception (Herek et al., 2009). Out of the perception of the stigmatization, the victim will have the corresponding emotional response, including identity misunderstanding, negative emotion arousing, health problems, and social dysfunction (Miller and Kaiser, 2001). Stigmas were traditionally perceived as controllable, with patients experiencing more anger and judgments neglected, contributing to complicated emotions, especially negative ones, children who encounter greater HIV stigma will experience more negative emotions (Wei et al., 2016). Several common emotional reactions include pity and anger (Weiner et al., 1988), fear or a sense of peril (Jones and Berglas, 1978), and sometimes even mental illnesses and cognitive coping responses predicting lower self-esteem and more hopelessness (Rüsch et al., 2009).

Stigmatization comes during widespread outbreaks of infectious disease, which contributes to stress (Goffman, 2009). The rejection perception may harm the well-being, psychological, and physical health of stigmatized groups (Ali et al., 2015). The perception of public stigma often leads to internalized stigma, resulting emotional and behavioral consequences, reduction of self-esteem (Corrigan et al., 2006), psychological distress (Corrigan et al., 2006), and withdrawal behaviors (Yanos et al., 2008). Observing the emotional response is important to understanding reactions to stigma, but the emotion influence of stigmatized people has not been cared sufficiently (Link et al., 2004).

Stress reactions predict a lot of negative behavior as well as negative emotion outcomes (Rüsch et al., 2009), such as pity, anger, and anxiety (Dijker and Koomen, 2003; Towler and Schneider, 2005; Goffman, 2009). Previous studies have found that anger may lead to abuse of people with intellectual disabilities, while pity is a signal to differentiate social identities (Link et al., 2004). Furthermore, communication with people with disabilities provokes more anxiety (Silván-Ferrero, 2008). In fact, reactions to the stigmatized are not always negative. People often manifest ambivalence, a mixture of positive and negative emotions, across a wide range of stigmas (Carver et al., 1977). Under some conditions, people may react more positively to a stigmatized group than to a non-stigmatized one (Carver et al., 1978).

The dual-process model is to understand individual psychological reactions to the stigmatization perception (Pryor et al., 2004). It is also used to describe the phenomenon that those who recognize stigma may be motivated to compensate for or overcome their initial prejudicial reactions. In the reactions to stigma, psychological systems will get involved in the adaption process of different social contexts. One process is reflexive and associative, which governs the initial reactions, and the other is rule-based and reflective, which governs subsequent reactions. The reflexive process relates to instinctive emotional reactions. As time passes, the corresponding psychological influence caused by this process will show a Poisson-distribution of radical rise, and then a steady fall. The rule-based process relates to attributional considerations and derived emotional reactions. In time, the corresponding psychological influence caused by this process grows steadily and surpasses the influence of the reflexive process (Pryor et al., 2004).

Stigmatization will arouse emotions and trigger the stress response or reaction mechanism. Due to the global nature of the COVID-19 pandemic, stigmatization has become a psychosocial phenomenon with a larger scope and more influence. At present, when worldwide public health is facing difficulties, studies on the social-emotional burdens caused by stigmatization have real-life significance, thus, it is important to test the existing theories against the background of this global public health and security crisis.

## Social Identity and Social Cooperation

Stigmatization is often connected to social identity processes (Link and Phelan, 2001; Bandura, 2004), and affects with mechanisms of discrimination, expectancy confirmation, and automatic stereotype activation, and indirectly with threats to identity (Major and O'brien, 2005). Social identity is described as the understanding that one belongs to a certain social group, which is also the process of social classification through which people view themselves as members of the same category (Stets and Burke, 2000). As for the social context, when one is recognized as the “Other,” or an outsider, the process can typify the stigmatized groups (Roberto et al., 2020). Accordingly, stigmatization will lead to an imbalance in people's cognition of social identity, and it is associated with negative feelings (Heise, 1989; Derks et al., 2008). The emergence of stigma conveys a demeaning social identity (Crocker et al., 1998), which becomes a special source of stress and brings psychological distress to stigmatized individuals (Major and O'brien, 2005). The social basis of self-identity makes the situation of stigmatized individuals problematic; they may perceive misunderstanding about their identity in the environment more often than others (Kaufman and Johnson, 2004).

Social Identity Theory (SIT) and its developments also offer a vision of people as being active in reacting to various challenges to their identity, and it systematizes those responses into the theory, incorporating the strategies of identity management involved in reacting to negative social identity (Blanz et al., 1998). The strategies are of three categories: individual mobility, social competition, and social creativity (Tajfel and Turner, 1979). Individual mobility occurs when personal status change, and usually implies strong behavioral consequences (Blanz et al., 1998). Typically, defining oneself as separate from other group members and as a unique individual who is not affected by the evaluations of the group (Ng, 1989) is considered individualization. One way to accomplish this is to compete for a better evaluation of the group; another is to compete for allocations of resources to get some favor for their own group (Blanz et al., 1998). Social creativity promotes the action of finding alternatives to change the cognitive parameters, which are usually classified as collective strategies (Tajfel, 1978). On the other hand, people may focus on competition; the stigmatized group members will improve their own status by improving the status of the in-group (Blanz et al., 1998).

How to improve the group's overall image depends on the group's collective efforts (Tajfel and Turner, 1979). Social cooperation exists in social competition and social creativity, and there is always social cooperation among in-group members. Within a social group, members will generate assimilation effects by drawing similarities, thus resulting in feelings like trust and affection (Brewer, 1996). Social identity defines our species, helping people to coordinate their relations but also impeding widespread cooperation (Bowles and Gintis, 2013). People will adopt different identity strategies on different occasions and make strategic adjustments based on the situation—between staying independent and integrating into a certain group (Smaldino, 2019). Altruistic disposition within the group plays a pivotal role in the formation of social cooperation (Sussman and Cloninger, 2011).

Altruistic behaviors refer to the behaviors made by individuals as the act of helping or benefiting others (Kurzban et al., 2015). When jointly confronting the unexpected outbreak of a public crisis, groups resort to collaborative behaviors on the grounds of social identification (Svedin, 2016). Moreover, the stigmatization of the group lays an environmental basis for them to form a collective. According to the stages of human evolution, strong reciprocity has been proven as a stable evolutionary strategy, and a small number of strong reciprocators could integrate a group into a kind of self-regarding type (Gintis et al., 2003). The cultural group selection theory also states that, when individuals' cooperative behavior is beneficial to the entire population, groups with a higher degree of cooperation will survive due to strong adaptability (Cavalli-Sforza and Feldman, 1981).

During public health crises, stigmatization tends to exert influences on a wider range of groups (Bagcchi, 2020), thereby creating an once-in-a-lifetime research background and scenario for probing the effects of stigmatization-influencing mechanisms on social identification as well as on social cooperation. Against the backdrop of the pandemic, the clarification of such an influencing mechanism is of great significance to public relations, international exchanges, public health management, and global cooperation in the fight against COVID-19.

## Research Model and Hypothesis

**Figure 1:**
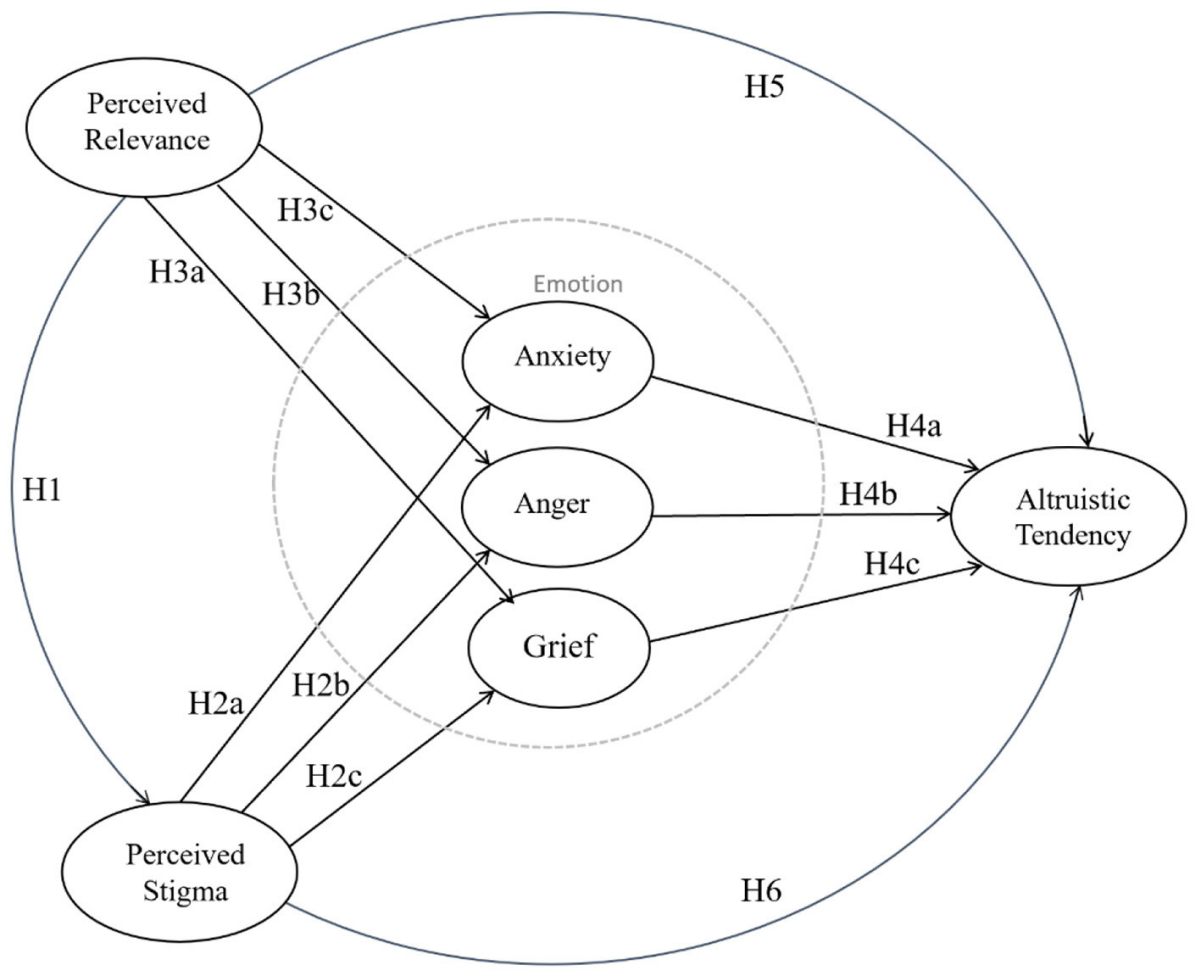
Research model.

We construct a model to examine how the factors of perceived stigma, perceived relevance, and negative emotion affect altruistic tendencies and online public stigmatization during the COVID-19 pandemic. The aim is to clarify negative emotion arousal and the altruistic behavior of public stigmatization during a pandemic and public health crisis facing people all over the world. The research model is depicted in Figure 1.

### Perceived Relevance

In the context of stigma, the specific social identity of the stigmatized individual is devalued among certain factions (Crocker et al., 1998), and such stigmatization often leads to discrimination against the stigmatized groups (Heatherton et al., 2000). Tajfel (1978) believes that people usually form their own social identity through the three basic psychological processes of social classification, social comparison, and active differentiation. In the stage of social classification, individuals divide the group into an inner group and an outer group, and establish connections between people and things in the inner group (Tajfel, 1982); that is, perceptual connection is one of the attributes of individual social identity. The correlation created by identity makes individuals attracted toward their own group. They adopt comparisons and differentiations to pursue positive social identity and focus on differences between groups, resulting in inter-group conflict and discrimination (Zhang and Zuo, 2006). In this case, the stigma from external group toward the members of internal group will provoke the stigmatization perception of the members belonging to internal group. Thus, we hypothesize that:

*H1: The perceived relevance of stigmatized groups has a positive impact on their perceived stigma during the COVID-19 pandemic*.

### Emotions: Anxiety, Anger, and Grief

Emotions are composed of many elements that are integrated into an affect program (Niedenthal and Ric, 2017). Stigma usually leads to negative emotions of the stigmatized, including stress, anxiety, sadness, and even some physical reactions (Lee and Craft, 2002; Armour, 2007; Lillis et al., 2020). Stigmatization is undoubtedly unfavorable to the victims and rubs salts into people's emotional wounds that exist as a result of the crisis. In particular, the public stigmatization of a certain region or group puts emotional burdens on its people and gives rise to their negative emotions (Bagcchi, 2020). These negative emotions are the integrated embodiment of several emotions: anxiety for suffering from pressure, anger for being offended, and sorrow for self-pity and depression (Corrigan and Watson, 2002; Griffiths et al., 2008; Zhu et al., 2017). Thus, we hypothesize that:

*H2a: The perceived stigma has a positive impact on the emotion of anxiety during the COVID-19 pandemic*.
*H2b: The perceived stigma has a positive impact on the emotion of anger during the COVID-19 pandemic*.
*H2c: The perceived stigma has a positive impact on the emotion of grief during the COVID-19 pandemic*.

According to the social identity process, people divide themselves as being inside a group or outside a group, establishing connections between people, concepts, and factors in the inner group (Tajfel, 1982). The concept of perceived relevance describes the intrinsic sources of personal relevance, or an intra-personal perception (Celsi et al., 1992). Group identification will induce the individual's perception of the events to become correlated with that of the group. The more individuals identify with the group, the stronger correlation they perceive between events and themselves (Henri and Turner, 1986). We argue that when a group suffers stigmatization, stronger perceptions of correlation will trigger stronger emotional experiences. Thus, we hypothesize that:

*H3a: The perceived relevance has a positive impact on the emotion of anxiety during the COVID-19 pandemic*.
*H3b: The perceived relevance has a positive impact on the emotion of anger during the COVID-19 pandemic*.
*H3c: The perceived relevance has a positive impact on the emotion of grief during the COVID-19 pandemic*.

### Altruistic Tendencies

Group emotions occur in and are shared with a collective of people at a moment in time, and both positive and negative emotions are affected by others (Niedenthal and Ric, 2017). In cyberspace, the stigmatization incidents and their ensuing negative emotions could easily spread among groups. The post-truth phenomenon omnipresent in cyberspace reflects the resonance of group emotions (McIntyre, 2018). The behaviors of assistance that are regarded as altruistic include acts motivated by shame or the willingness to maintain a positive self-image (Eisenberg, 2014). Compassion and empathy are the major emotions that are helpful for generating altruistic emotions (Hatfield et al., 2011). When it comes to a public health crisis, however, stigmatization introduces negative collective emotions that spread via networks to easily generate empathy (McIntyre, 2018). Once common feelings are awakened, collective empathies could boost the tendency for altruism (McAuliffe et al., 2018). As the world succumbed to the crisis of COVID-19, the collective stigmatization during the outbreak has made the stigmatized develop complex feelings, such as anxiety, anger, and sadness. Studies reveal that, in certain situations, an angry mood could raise attention toward fairness and justice as well as enhance cooperation tendencies and moral behaviors (Van Doorn et al., 2014). Thus, we hypothesize that:

*H4a: The emotion of anxiety has a positive impact on altruistic tendencies during the COVID-19 pandemic*.
*H4b: The emotion of anger has a positive impact on altruistic tendencies during the COVID-19 pandemic*.
*H4c: The emotion of grief has a positive impact on altruistic tendencies during the COVID-19 pandemic*.

The theory of social identity holds that individuals' identity with a group is the basis of group behavior. Through this group identity, individuals have a connection with the group, and the consciousness of belonging to a group will strongly affect our perceptions, attitudes, and behavior (Tajfel and Turner, 1979). In groups, people cooperate extensively with non-relative members (Gintis, 2000; Boyd et al., 2003), and perceived self-correlation will affect people's altruistic help choices, decision-making time, and subjective negative emotional responses (Zhan et al., 2019). Thus, we hypothesize that:

*H5: The perceived relevance of stigmatized groups has a positive impact on altruistic tendencies during the COVID-19 pandemic*.

Stigma often leads to stigmatization, prejudice, and discrimination against stigmatized groups (Dovidio et al., 2000). Therefore, stigma may directly affect the cognition, emotions, and behavior of the stigmatized individual (Miller and Major, 2000). The continuing threat of the current pandemic has increased stigma against China (Asmundson and Taylor, 2020; Bavel et al., 2020). People who regard themselves as stigmatized may confirm and disclose their identity out of intrinsic motivation (Swann, 1983; Ragins, 2008). When the corresponding social identity is negatively affected, individuals may use competition, collective behavior, and other positive behavior strategies to enhance the overall image of the group (Blanz et al., 1998). Individuals' cooperative behavior is beneficial to the entire population; and groups with a higher degree of cooperation will survive due to strong adaptability (Cavalli-Sforza and Feldman, 1981). Collectivism represents a strong tendency for individuals to cooperate (Wenninger et al., 2019). Therefore, we believe that Chinese groups with obvious collectivism tend to be more altruistic when they perceive stigma. Based on this, we hypothesize:

*H6: The perceived stigma of stigmatized groups has a positive impact on their altruistic tendency during the COVID-19 pandemic*.

### Mediating Effects of Emotion

Emotion is the physical and psychological response of information from the environment, which depends on people's evaluation of the information (Folkman and Lazarus, 1984). When an individual is faced with an unfavorable situation, they will first evaluate the threat, challenge, or degree of harm that the event or situation poses, and then produce a series of emotional reactions. For example, when people perceive hazards and threats in information, they produce negative emotions, such as anger, sadness, and anxiety (Folkman and Lazarus, 1984). Therefore, when people are stigmatized, they will think that they are being discriminated against and threatened based on the relevance of their identity and the perceived degree of stigma. According to the theory of resource conservation, when individual resources are threatened or lost, negative emotions, such as stress and anxiety, will be experienced (Shantz et al., 2016), and one's emotional state can affect their action tendency and behavior intention (Barnes et al., 2015). Some studies have confirmed the mediating role of emotion related factors in human behavior (Dennis et al., 2010; Karreman and Vingerhoets, 2012). That is to say, affection plays a mediating role between perception and behavior. Thus, we hypothesize that:

*H7: Negative emotions have mediating effects on the impact of perceived relevance on altruistic tendency*.
*H8: Negative emotions have mediating effects on the impact of perceived stigma on altruistic tendency*.

## Materials and Methods

**Figure 2:**
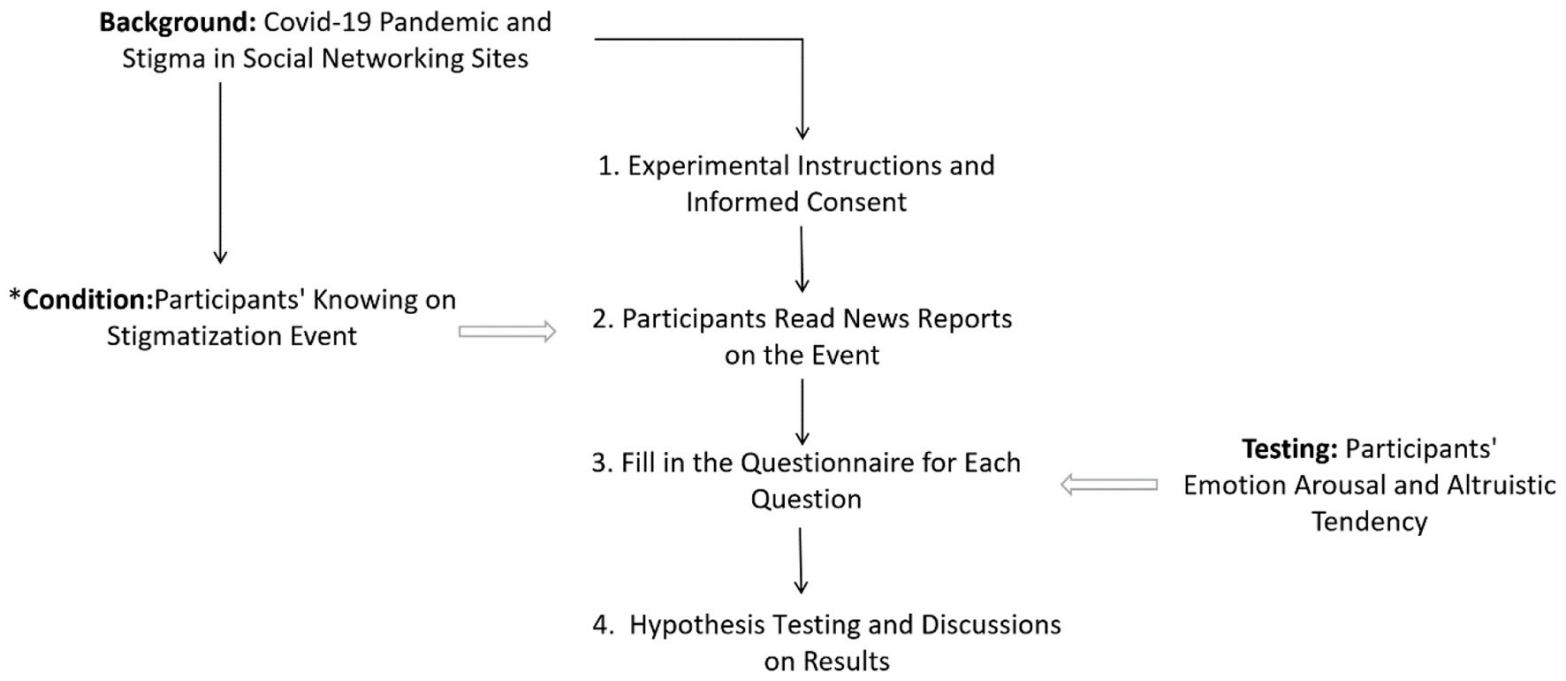
Research design and experimental process. * Is a prerequisite for carrying out the research as well as a factor to be controlled and tested.

Research design as well as the data collection for this study has been coincided with an important time point of pandemic prevention and control and has attracted widespread public attention. With the crisis sweeping the world, the traceability of the virus has become the focus of global attention. There is no evidence that this virus originated in any place all over the world. The earliest reported case in Wuhan had no history of contact with the seafood market (Huang et al., 2020), and the Wuhan seafood market may not be the origin of the novel coronavirus, SARS-CoV-2 (Cohen, 2020). However, some influential people publicized the stigma on his twitter account, which had hundreds of millions of followers. At the same time of the occurrence and fermentation of the stigma event, we carried out the design, development, and experimental data collection of the study. The experimental process is described in the Figure 2. It is very important to the participants' knowing of the event and to guarantee the authenticity and reliability of the data obtained, we mark this key factor in the Figure 2 with ^*^. The questionnaire employed consists of three parts. The first part is the privacy and protection statement and the informed consent statement. Participants first read and clicked the agreement option online. The second part is a news report, which has been summarized by two researchers based on real reports on the authoritative and influential official media. The report provided an objective description of the incident. The third part required participants to complete a questionnaire about the situation and their feelings; the questionnaire also tests the participants' understanding of stigma events and emotional arousal.

## Data Collection

The participants involved in this study are the Chinese group stigmatized in the event. The questionnaire was developed by referencing and adapting measurements from the literatures, and the hypothesis model proposed was verified by the data obtained. Before conducting the formal investigation, we employed a preliminary test based on a similar situation within the country during the pandemic. The pilot test included interviews and questionnaires to verify the results of the preliminary test stage and improve the research; then, a formal experiment was conducted.

The data were collected with a questionnaire using a sample service provided by an online survey platform (wjx.cn/sample/service.aspx).This is the largest online survey agency in China, providing 2.6 million sample banks consistent with the demographic distribution of China's netizen. As one of the current typical academic research methods, online survey has been widely recognized for its advantages of timeliness, maneuverability and so on (Evans and Mathur, 2018). Although its representativeness has been questioned, scholars believe that when most people in a society have Internet access and savvy, the basic feedback of using online research-the lack of representativeness-will lost (Scholl et al., 2002). By December 2020, the number of Internet users in China reached 989 million, and the Internet penetration rate reached 70.4 percent (CNNIC, 2021), furthermore, as this study needs to involve situational control and eliminate possible bias effects, online investigation is well-applicable to our study (Evans and Mathur, 2005). The survey and data collection mainly included three processes. First, participants were required to read and confirm the informed consent instructions and to complete the survey. They were told that the data were only to be used for scientific research, without influencing their privacy, reputation, living conditions, or health. Second, participants completed the survey. Finally, they received a lottery ticket after completing the questionnaire.

Before administering the questionnaire, the participants were asked to read a news report description and fill out the online survey based on their understanding of this event. To ensure the quality of questionnaire and prevent the occurrence of repeated surveys by participants, the questionnaire was set to be answered based on each user's social media account, which could only be retrieved once. We used the time limit as a screening factor. The questionnaire was considered invalid if the time spent was <3 min, and two reverse-logic questions were included in different positions of the questionnaire. All answers that violated the reverse-logic setting were considered invalid. We used AMOS for the empirical analysis. AMOS can be used for covariance-based structural equation model analysis (CB-SEM), which is accepted and used by a growing number of researchers as user-friendly statistical software (Hair et al., 2014).

## Measurements

We combined the existing theories and related literature, put forward theoretical models and hypotheses, summarized the latent variables that needed to be measured, and adapted them to form the measured variables and specific items according to the existing literature. The grief scale and altruistic tendency scale have developed for this research to measure grief and altruistic tendency in response to the stigmatization of the event, these items loaded on a single factor with no factor loadings below 0.75. item responses were averaged to create a single index of grief (Cronbach's α = 0.934; CR = 0.936; AVE = 0.785), as well as altruistic tendency (Cronbach's α = 0.903; CR = 0.906; AVE = 0.707).

To adapt to the understanding of Chinese users and avoid the problem of misunderstanding caused by language differences, we translated the scale into Chinese and then into English to verify the consistency of the expressions in the scale and to ensure that the translation and expressions are consistent. A five-point Likert scale (1 = completely disagree, 5 = completely agree) was used for measurement. The structures and measures used in this study and the source references are listed in **Table 2**, as well as the Cronbach's α values, composite reliability (CR), and average variance extracted (AVE) of the constructs, as well as the loading, *T*-value, mean, and standard deviation (SD) of the measured items.

In this study, SEM was used to test the hypotheses, and covariance analysis was used for the statistical analysis. We used SPSS 25.0 and AMOS to carry out the empirical analysis of the obtained survey data. This not only helps to solve the relationship problem of multiple dependent variables, but also can test the relationships between many potential structures by reducing model errors (Hair et al., 2014). Meanwhile, CB-SEM applications also contribute to scale development, exploratory and confirmatory analysis, relative saliency of potential structures, and assessment of causality (Hair et al., 2010; DeVellis, 2011). We tested the reliability and validity of the data to ensure the availability of the data and the validity of subsequent conclusions, and then evaluated the structural model. The specific reliability test results are shown in **Table 2**.

## Results

We collected 365 questionnaires, including 313 valid questionnaires. Table 1 presents the survey results on the characteristics of the respondents. The representativeness of the sample is reasonable. The distribution of male and female participants is in accordance with the gender distribution of Internet users in China.

**Table 1:** Descriptive statistics of participants' characteristics.

**Characteristic**		**Number (%)**
Gender	Female	150 (47.9)
	Male	163 (52.1)
Age (years)	Under 18	1 (0.3)
	18–25	257(82.1)
	26–30	8 (2.6)
	31–40	34 (10.9)
	41–50	12 (3.8)
	51–60	1 (0.3)
Familiarity with the event	Quite familiar	26 (8.3)
	Relatively familiar	171 (54.6)
	Neutral	94 (30.0)
	Relatively unfamiliar	18 (5.8)
	Unfamiliar	4 (1.3)
Education level	Primary school and below	0 (0)
	Junior middle school	1 (0.3)
	Senior middle/Technical secondary school	19 (6.1)
	Junior college	10 (3.2)
	Bachelor's degree	210 (67.1)
	Master's degree or above	73 (23.3)
Visiting abroad	Yes	54 (17.3)
	No	259 (82.7)

## Reliability and Validity

The composite reliability (CR) and internal consistency (Cronbach's alpha) of latent variables are usually used as important indicators for evaluating model reliability. Average Variance Extracted (AVE) is usually used to measure convergence validity. Table 2 shows the α coefficient, CR value, and AVE of each latent variable, as well as the loading, *T*-value, mean, and SD of the measured variables. Except for the removed AG2, and AT5, the two items with loading values <0.6, the factor loadings of all measurement indicators are >0.6, and most are >0.8, indicating that they measure their respective latent variables well. This also ensures the better convergence of the measurement model. In addition, the AVE values of all latent variables are >0.6, and most are >0.7, indicating that the latent variables have good convergence validity.

**Table 2:** The measures and psychometric properties.

**Items**	**Loading**	***T*-Value**	**Mean**	**SD**
**Perceived relevance Kalyanaraman and Sundar, 2006 (Cronbach's α = 0.886; CR = 0.888; AVE = 0.615)**				
PR1: This event is important to me	0.837	53.572	3.350	1.105
PR2: This event makes sense to me	0.900	62.143	3.480	0.991
PR3: I care about the impact of this incident	0.803	63.377	3.720	1.039
PR4: I can relate this to my experience	0.651	57.350	3.190	0.983
PR5: The result of this event is relevant to me	0.705	57.452	3.340	1.029
**Perceived stigma Pinel and Paulin, 2005 (Cronbach's α = 0.902; CR = 0.907; AVE = 0.665)**				
PS1: Our behavior is influenced by prejudice	0.716	55.334	3.470	1.109
PS2: People from other countries will have negative thoughts about us even if they don't express them	0.935	72.181	3.790	0.928
PS3: It is difficult for people from other countries to treat us equally because of the prejudice that this produces	0.916	68.459	3.620	0.936
PS4: People from other countries will look at us in an unequal way because of the prejudice that produces	0.799	66.642	3.560	0.946
PS5: People from other countries will be reluctant to deal with us because of the prejudice	0.678	54.753	3.120	1.009
**Anxiety Kay and Loverock, 2008 (Cronbach's α = 0.886; CR = 0.899; AVE = 0.640)**				
AX1: When I found out about the incident, I was upset	0.802	59.559	3.190	0.947
AX2: I'm afraid something bad will happen after this incident	0.705	63.456	3.470	0.967
AX3: When I think of this incident, I feel anxious and uneasy	0.879	60.452	2.990	0.875
AX4: When I saw or heard the online/side dispute about the matter, I felt nervous and could not relax	0.814	55.466	2,910	0.927
AX5: I worry about the development of this matter	0.791	59.567	3.250	0.965
**Anger Vassilikopoulou et al., 2011 (Cronbach's α = 0.898; CR = 0.900; AVE = 0.695)**				
AG1: I was very annoyed at the incident	0.829	72.424	3.730	0.910
AG2: I'm tense about this. (dropped)				
AG3: I want to shout about the incident	0.728	69.617	3.400	0.865
AG4: I feel angry about the incident	0.919	71.323	3.680	0.913
AG5: When I found out about it, I felt angry	0.846	65.651	3.430	0.925
**Grief Cohen and Hoffner, 2016 (Cronbach's α = 0.934; CR = 0.936; AVE = 0.785)**				
GF1: I feel very sad about the incident	0.815	60.167	3.230	0.949
GF2: I feel very depressed about the incident	0.877	59.409	3.120	0.931
GF3: The incident made me sad	0.941	60.038	3.170	0.935
GF4: The incident made me feel very sad	0.906	59.543	3.200	0.950
**Altruistic tendency Kurzban et al., 2015 (Cronbach's α = 0.903; CR = 0.906; AVE = 0.707)**				
AT1: I will not hesitate to help others	0.792	85.681	3.620	0.747
AT2: If I had a chance, I'd be happy to help others	0.838	103.040	3.940	0.677
AT3: I will sincerely care about the difficulties of others	0.912	102.386	3.860	0.666
AT4: I will appeal to people around me to help others	0.817	93.267	3.720	0.705
AT5: After this incident, I was unwilling to help others from the bottom of my heart. (dropped)^*^				

* *Reversed scale*.

According to the Fornell-Larcker Criterion, when the square root of the AVE of a variable is greater than its correlation coefficient with a certain variable, the two variables have good discriminant validity. Table 3 shows that all the values on the diagonal are >0.7 and greater than the values under the diagonal, indicating that the value of the square root of the AVE of all variables is greater than the correlation coefficient between the variables; therefore, the discriminant validity between all variables is acceptable.

**Table 3:** Correlation matrix and psychometric properties of key constructs.

	**PR**	**PS**	**AX**	**AG**	**GF**	**AT**
Perceived relevance (PR)	0.784					
Perceived stigma (PS)	0.365	0.815				
Anxiety (AX)	0.447	0.320	0.800			
Anger (AG)	0.397	0.418	0.533	0.834		
Grief (GF)	0.272	0.202	0.524	0.411	0.886	
Altruistic tendency (AT)	0.197	0.278	0.295	0.447	0.310	0.841

*SQRT (AVE) is in parentheses. Off-diagonal cells show the correlations between constructs*.

## Structural Model

**Figure 3:**
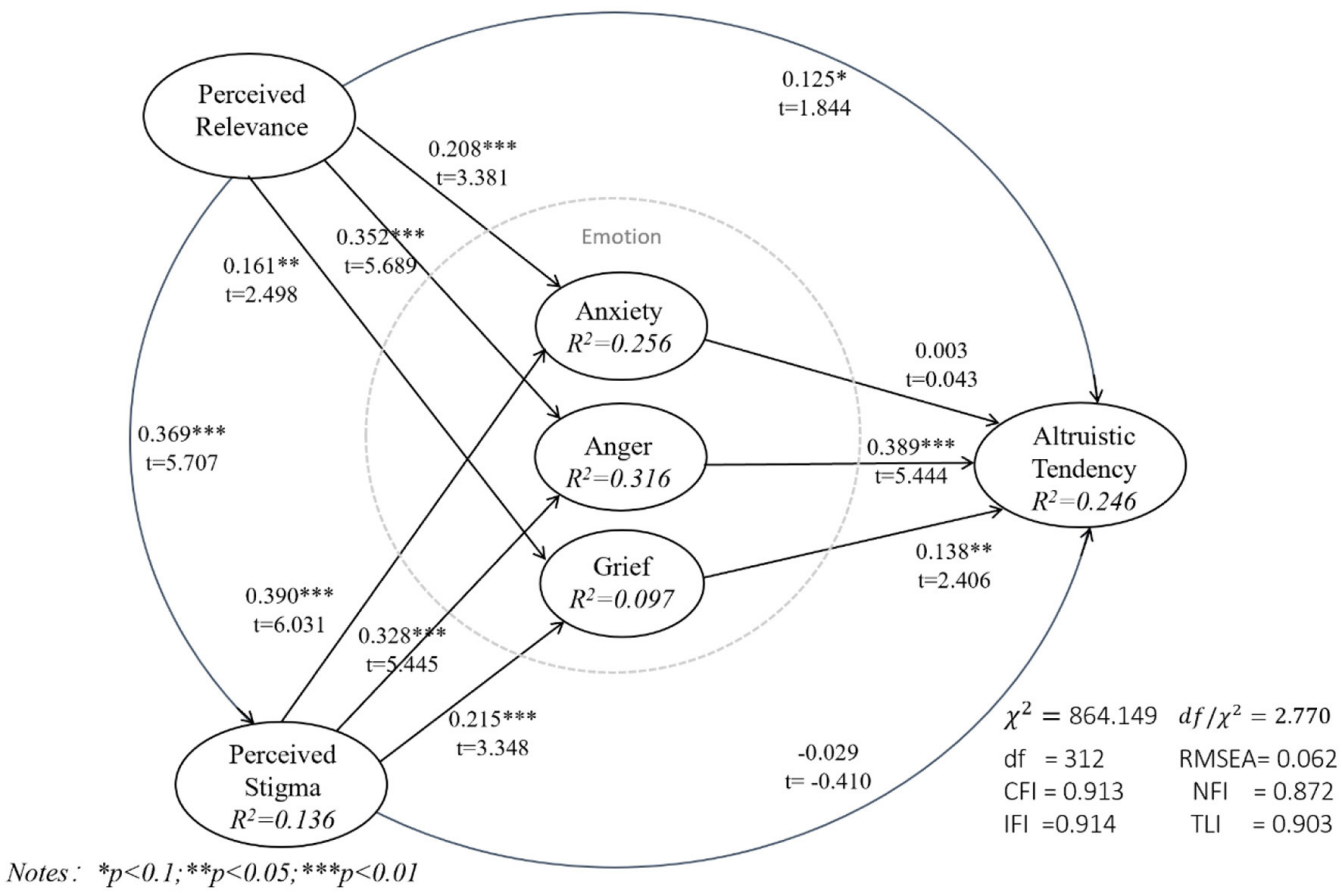
Results of the research model.

This study uses AMOS to test the constructed model by analyzing the path coefficient, the significance of the coefficient, the determination coefficient *R*^2^, and the fitness index of the model. Before testing the hypotheses, the multicollinearity of the relevant data structure has been tested and meets the requirements. Figure 3 shows the path between each construct, the path coefficient, the corresponding *T*-value of the coefficient and its significance to the structural equation model, and the corresponding *R*^2^ results.

For the complete model, the test results confirmed most of the hypotheses proposed in this study. Except for H4a and H6, the hypotheses are strongly supported by empirical evidence, with a *p* < 0.05, and most hypotheses have a *p* < 0.01. This is strong support from the empirical evidence. Regarding perceptual relevance, we found that relevance has a strong positive effect on the stigma of perception (β = 0.369, *p* < 0.01). This finding is consistent with the results of previous studies. The occurrence of this incident evokes the identity perception of the Chinese public, extending itself and combining with the whole country, the higher the degree of association, the higher the degree of perceived stigma. Therefore, H1 is supported.

The perceived stigma significantly affected participants' anxiety (β = 0.390, *p* < 0.01), anger (β = 0.328, *p* < 0.01), and grief (β = 0.215, *p* < 0.01). Therefore, H2a, H2b, and H2c are supported. The results show that relevance significantly leads to people's negative emotions, including anxiety (β = 0.208, *p* < 0.01), anger (β = 0.352, *p* < 0.01), and grief (β = 0.161, *p* < 0.05); in the stigmatized scene, the higher the relevance of the participants, the more likely they were to be aroused into negative emotions. Therefore, H3a, H3b, and H3c were verified. In addition, the coefficient of the path from relevance to anger is greater than the coefficient of the path from relevance to anxiety and grief, indicating that anger with a high-relevance perception dominates the negative emotions.

As for the effect of emotions, the effect of anxiety on altruistic tendency is not significant (β = 0.003, *p* = 0.966), so H4a is not supported. Anger (β = 0.389, *p* < 0.01) and sadness (β = 0.138, *p* < 0.05) significantly and positively promote participants' altruistic tendency, supporting H4b and H4c. The coefficient of the path from anger to altruism is significantly greater than that from grief to altruism, providing further evidence that, during the COVID-19 pandemic, negative emotions caused by people being stigmatized have a pro-altruistic effect on the stigmatized subjects. Moreover, though anger occupies the dominant position, the impact of anxiety cannot be verified. In addition, perceived relevance also promotes the altruistic tendency to a certain extent (β = 0.125, *p* < 0.1), therefore, H5 is supported. However, perceived stigma has no significant impact on altruistic tendency, thus, the empirical evidence does not support H6.

The independent variables explain a substantial portion of the variance in the dependent variables. Perceived relevance explains 13.6% of the variance in perceived stigma, 25.6% of the variance in anxiety, and 31.6% of the variance in anger are explained by perceived relevance and perceived stigma. Although the explained variance portion of grief is relatively low (9.7%), the model accounts for 24.6% of the variance in altruistic tendency.

Furthermore, we report the model fitness indicators listed in Figure 3, which are widely used in SEM testing. As suggested by Marsh and Hocevar (1985), when *df* / χ^2^ is between 1 and 3—the value here is 2.770—the root mean square error of approximation (RMSEA) should be <0.08—here, it is 0.062. When the values of certain indicators in NFI, IFI, TLI, and CFI are >0.9, this indicates a good fit to the data, and the fit indicators reported here suggest that the model has reasonable fit.

Based on the analysis of the direct effect (DE), indirect effect (IE), and total effect (TE) of the model constructs presented in Table 4, we summarized the mediating effects of the emotions. Perceived relevance has a significant TE on altruism tendency (TE = 0.223, *p* = 0.002), so the follow-up analysis can be carried out according to the mediating effect (Wen and Ye, 2014). The DE from perceived relevance to altruistic tendency is not significant (DE = 0.098, *p* = 0.150), while the total indirect effect (TIE) from perceived relevance to altruistic tendency is significant (TIE = 0.044, *p* = 0.000), indicating that the impact from perceived relevance to altruistic tendency is completely mediated by negative emotions; therefore, H7 is supported. As for the factors between perceived relevance and altruistic tendency, the IE of anxiety is not significant (IE = 0.015, *p* = 0.960), while the IEs of anger (IE = 0.037, *p* = 0.000) and grief (IE = 0.014, *p* = 0.057) are significant, accounting for 85.6 and 14.4% of the IEs, respectively.

**Table 4:** Direct, indirect, and total effect (Bootstrap = 2,000).

**Effect types**		**Effect mean**	**SE**	**95% CI**		**P**
				**Lower**	**Upper**	
Total effect	PR → AT	0.223	0.076	0.088	0.387	0.002
	PS → AT	0.102	0.072	−0.038	0.243	0.144
Direct effect	PR → AT	0.098	0.073	−0.035	0.255	0.150
	PS → AT	−0.022	0.063	−0.143	0.108	0.731
Total indirect effect	PR → AT	0.125	0.044	0.058	0.238	0.000
	PS → AT	0.124	0.047	0.049	0.233	0.001
Indirect effect	PR → AX → AT	0.000	0.015	−0.034	0.030	0.960
	PR → AG → AT	0.107	0.037	0.051	0.203	0.000
	PR → GF → AT	0.017	0.014	0.000	0.059	0.057
	PS → AX → AT	0.001	0.025	−0.047	0.052	0.983
	PS → AG → AT	0.100	0.035	0.044	0.184	0.000
	PS → GF → AT	0.023	0.014	0.004	0.064	0.019

Perceived stigma has no significant TE on altruism tendency (TE = 0.072, *p* = 0.144), but the DE from perceived stigma to altruistic tendency is not significant (DE = 0.063, *p* = 0.731), while TIE from perceived relevance to altruistic tendency is significant (TIE = 0.047, *p* = 0.001); thus, the follow-up analysis should be carried out according to the suppressing effect (Wen and Ye, 2014). However, the total mediating effect of emotions between perceived stigma and altruism tendency also exist under this situation (Fairchild and McQuillin, 2010; Rucker et al., 2011; Soest and Hagtvet, 2011), indicating that the impact from perceived stigma to altruistic tendency is completely mediated by negative emotions; thus, H8 is supported. As for the factors between perceived stigma and altruistic tendency, the IE of anxiety is not significant (IE = 0.001, *p* = 0.983), while the IEs of anger (IE = 0.100, *p* = 0.000) and grief (IE = 0.023, *p* = 0.019) are significant, accounting for 81.3 and 18.7% of the IEs, respectively.

## Discussion

In the course of forming social identity, people assign themselves to different groups by judging and evaluating their own and others' affiliations. This is done to divide the group into an inner group and an outer group, and establish connections between people and things in the inner group (Tajfel, 1982). Perceived relevance is one of the attributes of social identity. However, there are discrimination and conflicts among social groups (Zhang and Zuo, 2006). In the case of group stigmatization, individuals with an intense sense of group belonging will experience a higher perceived level of being stigmatized. Therefore, the support of H1 in the current study supplies evidence for the social identification mechanism of the generation of perceived stigma in the context of public stigmatization.

With the supportive results for H2a, H2b, and H2c, public stigmatization has been shown to have a significant negative effect on emotions arousal during the COVID-19 pandemic. The stronger the perceived level of stigma by the stigmatized person, the more anxiety, anger, and grief they will have; this is consistent with the findings of other studies on negative emotions caused by stigma in other scenarios (Lee and Craft, 2002; Armour, 2007; Bagcchi, 2020; Lillis et al., 2020). Among the pathways of influence in which perceived stigma evokes negative emotions, anxiety has the largest pathway coefficient, followed by anger and then grief, which has the smallest coefficient, but one that is still significant.

Furthermore, the perceived relevance caused by social identity significantly promotes negative emotions of the public stigma of COVID-19, as shown by the supportive results for H3a, H3b, and H3c. This is consistent with the negative effects of stigmatization in other social situations (Jones and Corrigan, 2014). The emergence of group identity is the antecedent condition for the perception of being stigmatized as a member of this group. This study further confirmed that perceived relevance among groups can significantly promote negative emotions in public stigma events. Among the pathways of influence in which perceived relevance evokes negative emotions, anger has the largest pathway coefficient, followed by anxiety and then grief, which has the smallest coefficient, but one that is still significant.

An interesting finding of this study is that negative emotions can promote social altruistic tendency in online public stigma about the COVID-19 pandemic. Although the impact of anxiety on altruism tendency is not significant, failing to support H4a, with the support for H4b and H4c, the current study confirmed the dominant role of anger and the significant role of grief in the promotion of altruism (Seip et al., 2014; Van Doorn et al., 2014). The findings confirmed that, facing the current crisis, the anger and grief produced by the members of the stigmatized group have a certain role in promoting their mutual help. In addition, perceived relevance also promotes the altruistic tendency, with supportive evidence for H5, verifying the promoting altruism effect of relevance perception in social identity (Gintis, 2000; Boyd et al., 2003; Zhan et al., 2019).

We found that negative emotions generated by stigmatized play a complete mediating effect between perceived relevance and altruistic tendency and a complete mediating effect between perceived stigma and altruistic tendency. As H6 was not supported and the total effect between the perceived stigma and altruism tendency was not significant, the role that emotions played was also referred to as the suppressing effect (Wen and Ye, 2014). The mediating effect of negative emotions is verified through emotions of anger and grief; specifically, anger takes a leading role, with supportive evidence for H7 and H8. As such, negative emotions play a mediating effect between social responses of stigmatizing and stigmatized groups. Perceived stigma and social identification induce responses of negative emotions, which in turn promote the tendency of altruistic tendency.

## Theoretical Implications

Our findings have several theoretical implications. First, our findings confirmed that public stigmatization behavior can significantly induce negative emotions in stigmatized groups, and it provides empirical evidence that this causes negative emotions including anxiety, anger, and grief. It lays a foundation for further exploration of the influence mechanism of media expression on public sentiment in a global public health crisis. Second, the current study clarified the social identity, stigma, and its evoking effect on negative emotions in the context of the COVID-19 pandemic. Negative emotions are a burden on society, and the present findings provide empirical evidence for the harm of stigma to social groups. From the perspective of public health management, the widespread production of negative emotions is undoubtedly a threat. Third, the present research is a beneficial attempt to apply and expand the study of social identity theory in the context of a public health crisis, which provides a basis for further expanding the interpretation of social identity theory in the same setting. On one hand, group perception association will strengthen stigma perception and aggravate the arousal of negative emotions. On the other hand, perceptual relevance can promote altruistic tendencies within groups, thus bringing about better cooperation conditions. These interesting conclusions are worth explaining and exploring based on more research scenarios. Fourth, the current study reveals that anger and grief caused by stigma can promote social altruistic behavior in the context of a public health crisis, and play an intermediary role between stigma and altruism. This discovery provides a basis for further research on the formation of social cooperation and the strategy of social collective mobilization when humans face public crisis events.

## Practical Implications

The conclusions presented here can provide certain inspiration for public expressions and international cooperation, and even the formation of the protest cooperative relationship in public health crisis events. First, we should not simply connect countries, regions, and groups directly with the outbreak, for this stigmatizing behavior will harm the stigmatized groups. Large-scale negative emotions (e.g., anxiety, anger, sorrow) are adverse to the maintenance of a positive attitude of the public. In the long run, possible threats can be posed to people's physiological health. Second, we should be cautious with our words and actions in the Internet public sphere. Today, with humans connected in a community of common fate via the Internet, the words and deeds of anyone could suddenly arouse wide concern and, thus, influence the world. In the context of the outbreak, with every corner of the world gripped by anxiety, the communication power of the Internet will expand the harm of stigmatization to others. Therefore, we should not make baseless accusations against others via social media. Third, we should not easily blame others for no reason, even the possible antagonists during a certain period. According to this research, the behaviors that evoked negative emotions promoted cooperation within the stigmatized. One might wish to pass the buck but end up helping others unintentionally.

## Limitations and Future Directions

First, the research scenario and participants in the survey were restricted to one country based on a typical stigma event during the COVID-19 pandemic. Additional research will be needed to examine how and to what extent contextual differences affect emotions and altruism tendency. The research model proposed is applicable in other social public crises and is capable of considering other factors related to public health. Second, this study focuses on the impact of online public stigma on social networking sites, we try to restore the scene of stigmatization event through online experiments, and although online questionnaires are widely used in behavioral research, it still has limitations. In future research, we consider to reconfirm the negative emotion arousal and altruism promoting of online public stigmatization by means of field interviews or offline experiment based on scenario setting. Third, some characteristics of the sample may cause the results to be biased—for example, respondents aged 18–25 accounted for 82.1% of the total, and those with a bachelor's degree or above accounted for 89.4% of the total. However, the altruistic behavior studied in this article is a strategy for human evolution and stability, which is a general problem of human behavior (Gintis et al., 2003), so the analysis is not limited by the demographic characteristics of the sample. This has been confirmed in related studies (Feng et al., 2020). Fourth, the influence of stigma on social groups differs within and outside of the group. This research examines the influence of public stigma on the stigmatized group. Future research should further verify the influence of public stigma on other groups in social public crisis events and deeply explore different types of emotional arousal mechanisms for different groups. Fifth, this research includes three typical negative emotions related to stigmatization. More emotions shall be taken into future research to systematically consider the aroused mechanism of different emotions and the impact on social cooperation tendency.

## Conclusion

The global public health crisis triggered by COVID-19 is still proceeding, and stigma has brought uncertainties to the prevention and control of the pandemic. In this study, we verified that, in the context of the COVID-19 pandemic, perception relevance and perception stigma have positive impacts on the negative emotions of people, and the arousal of negative emotions leads to a rise in the altruistic tendency within the stigmatized group, to a certain extent. The measurement model has been confirmed, with acceptable credibility and validity, path coefficients, and model fit. The results contribute to extend the knowledge on the negative emotion reactions on the public online stigma during the COVID-19 pandemic. For a country and nation, external misunderstanding and stigma will promote the group becoming more united and mutual help. One wish to pass the buck but end up helping others unintentionally.

## Data Availability Statement

The original contributions presented in the study are included in the article/Supplementary Material, further inquiries can be directed to the corresponding author/s.

## Ethics Statement

Ethical review and approval was not required for the study on human participants in accordance with the local legislation and institutional requirements. Written informed consent for participation was not required for this study in accordance with the national legislation and the institutional requirements.

## Author Contributions

XC performed the theory analysis and contributed to drafting the manuscript. CH performed the design and analyzed the data and the empirical analysis and contributed to drafting the parts of manuscript. HW and WW collected and analyzed the data and improved the writing. XN contributed to proof read the manuscript and funding. YL improved the writing and conclusions. All authors contributed to the article and approved the submitted version.

## Conflict of Interest

The authors declare that the research was conducted in the absence of any commercial or financial relationships that could be construed as a potential conflict of interest.
